# Dissolution Enhancement of Rosuvastatin Calcium by Liquisolid Compact Technique

**DOI:** 10.1155/2013/315902

**Published:** 2013-02-27

**Authors:** V. J. Kapure, V. V. Pande, P. K. Deshmukh

**Affiliations:** Department of Pharmaceutics and Quality Assurance, H. R. Patel Institute of Pharmaceutical Education and Research, Shirpur, Maharashtra 425405, India

## Abstract

In present investigation liquisolid compact technique is investigated as a tool for enhanced dissolution of poorly water-soluble drug Rosuvastatin calcium (RVT). The model drug RVT, a HMG-Co A reductase inhibitor was formulated in form of directly compressed tablets and liquisolid compacts; and studied for in-vitro release characteristics at different dissolution conditions. In this technique, liquid medications of water insoluble drugs in non-volatile liquid vehicles can be converted into acceptably flowing and compressible powders. Formulated systems were assessed for precompression parameters like flow properties of liquisolid system, Fourior transform infra red spectra (FTIR) analysis, X-ray powder diffraction (XRPD), differential scanning calorimetry (DSC), and post compression parameters like content uniformity, weight variation, hardness and friability, disintegration test, wetting time, *in vitro* dissolution studies, effect of dissolution volume on drug release rate, and estimation of fraction of molecularly dispersed drug in liquid medication. As liquisolid compacts demonstrated significantly higher drug release rates, we lead to conclusion that it could be a promising strategy in improving the dissolution of poor water soluble drugs and formulating immediate release solid dosage forms.

## 1. Introduction

The poor dissolution rate of water-insoluble drugs is still a substantial problem confronting the pharmaceutical industry. A great number of new and, possibly, beneficial chemical entities do not reach the public merely because of their poor oral bioavailability due to inadequate dissolution [1, 2].

Over the years, various formulation techniques like the formation of water-soluble molecular complexes, drug micronization, solid dispersion, coprecipitation, lyophilisation, and microencapsulation are some of the major formulation tools which have been shown to eknhance the dissolution characteristics of water-insoluble drugs [3, 4]. The liquisolid compacts are a new and promising addition towards such a novel aim. This technique is a compressible and free-flowing powdered form of liquid medication. Using this new formulation technique, a liquid medication may be converted into a dry-looking, nonadherent, free-flowing, and readily compressible powder by a simple blending with selected powder excipients referred to as the carrier (cellulose, starch, lactose, etc.) and coating (silica) materials [5–7]. Research shows that soft gelatin capsules contain a solubilised liquid drug with a higher and more consistent bioavailability than the conventional oral dosage forms, due to the active ingredient present in the solution. Liquisolid compact delivers active ingredients in form of soft gelatin capsule preparation which contains liquid; where non-volatile solvent is used to dissolve the solid drug. Therefore, the drug is held in the solution, even though it is in a tabletted or encapsulated dosage form. Consequently, drug dissolution properties will be improved [8–10]. 

The present work deals with formulation of liquisolid compact of RVT calcium, a poor water-soluble drug, and its evaluation for precompression and postcompression parameters. The effect of dissolution media and their volume on the *in vitro* drug release rate was also studied.

*Theoretical Aspects*. In the fundamental studies made by Spireas et al. [8, 9], flow and compression issue have been addressed with the use of the new mathematical model of liquisolid system which is based on the flowable (*ϕ*-value) and compressible (*ψ*-number) liquid retention potentials of the constituents powder. The good flow and compression properties of the liquisolid system are encouraged by the large surface area and fine particle size. 

According to the new theories, the carrier (*Q*) and coating (*q*) powder materials can retain only certain amount of liquid while maintaining acceptable flow and compression properties. Depending on the excipients ratio (*R*) or the carrier: coating ratio of the powder system used, where

(1) $$\begin{array}{c} R=\frac{Q}{q}. \end{array}$$

  (*R*) represents the ratio between the weights of carrier (*Q*) and coating (*q*) materials present in the formulation. A free-flowing and compressible liquisolid system can be prepared only if the maximum liquid on the carrier material is not exceeded; such a characteristic amount of liquid is termed as the liquid load factor (*L*_*f*_) and defined as the ratio of the weight of the liquid medication (*W*) over the weight of the carrier powder (*Q*) in the system, which should be possessed by an acceptably flowing and compressible liquisolid system [10, 11]. That is,

(2) $$\begin{array}{c} L_{f}=\frac{W}{Q}. \end{array}$$

The flowable liquid retention potentials (*ϕ*-value) of powder excipients are considered to calculate the required ingredient quantities [8]. Hence, the powder excipients ratios *R* and liquid load factors *L*_*f*_ of the formulations are related as follows:

(3) $$\begin{array}{c} L_{f}=\phi +\phi \left(\frac{1}{R}\right). \end{array}$$

In order to calculate the required weights of the excipients used, first, from (3), *ϕ* and *ϕ* are constants; therefore, according to the ratio of the carrier/coat materials (*R*), *L*_*f*_ was calculated from the linear relationship of *L*_*f*_ versus 1/*R* [11]. Different weights of the liquid drug solution (*W*) will be used depending on the liquid vehicle concentration; with the knowledge of *L*_*f*_ and *W*, the appropriate quantities of carrier (*Q*_*o*_) and coating (*q*_*o*_) powder materials required to convert a given amount of liquid medication (*W*) into an acceptably flowing and compressible liquisolid system could be calculated from (1) and (2).

## 2. Experimental

### 2.1. Materials

RVT was obtained as a gift sample from Cadila pharmaceuticals Ltd., Ahmadabad. Aerosil 200 was procured from Research Lab, Avicel PH 102 and propylene glycol were purchased from Loba Chemicals Mumbai, polyethylene glycol 400, Tween 80, and sodium starch glycolate were purchased from Merck. All chemicals and reagent, and used were of analytical grade.

### 2.2. Methodology

#### 2.2.1. Solubility Studies

The solubility of RVT in water and the three liquid vehicles which were used to prepare the liquisolid system, namely, propylene glycol, polyethylene glycol 400, and Tween 80, were studied by preparing a saturated solution of the drug in these solvents and their drug content was analysed spectrophotometrically. Also, rosuvstatin, was mixed with each of the previous solvents in 10 mL glass vial individually in such quantity so as to produce systems containing an excess of drug. The mixtures were sonicated for 24 h and then cooled down to 25°C under constant vibration. Accurately weighed quantities of the filtered supernatant solutions were further diluted with methanol after centrifugation and analyzed spectrophotometrically at 242.6 nm for their drug content. The results were extrapolated to determine the percent w/w of RVT in its saturated solution with the solvent under investigation.

#### 2.2.2. Preparation of Directly Compressed Tablets

A conventional formulation of RVT was directly compressed into cylindrical tablets, each containing 10 mg drug. In addition, each tablet contained the following powder excipients: 140 mg coarse granular microcrystalline cellulose (Avicel PH 102), 70 mg lactose monohydrate, 10 mg Aerosil 200, and 20 mg sodium starch glycolate. Twenty tablets batches were mixed in a mortar for 10 min and compressed using a compression machine. Sufficient compression loads were applied in order to produce tablets of 3–5 kg/cm^2^ hardness.

#### 2.2.3. Preparation of Liquisolid Compacts

Several liquisolid systems of RVT were prepared in 20 tablet batches and compressed into cylindrical tablets of 10 mg strength each, using compression machine and a target hardness of 3–5 kg/cm^2^. All liquisolid formulations contained microcrystalline cellulose as the carrier powder and silica as the coating (covering) material at a fixed powder excipient ratio (*R*) of 20. Propylene glycol was used as the liquid vehicle to prepare the liquid medications of different drug concentrations, ranging from 10 to 20% w/w, included in the formulations LST-1 to LST-3. On the other hand, PEG 400 was used as the liquid vehicle in the formulations LST-4 to LST-6, and, similarly, Tween 80 was used as the liquid vehicle in the formulations LST-7 to LST-9 with similar drug concentration. Depending on the liquid vehicle and drug concentration in the liquid medication used, different liquid load factors (*L*_*f*_) ranging from 0.195 to 0.235 (w/w) were employed in liquisolid preparations. Finally, a standard 5% (w/w) of the disintegrant sodium starch glycolate was added to all liquisolid systems. Important formulation characteristics of RVT liquisolid compacts are shown in Table 1.

### 2.3. Application of Mathematical Model for RVT Liquisolid System

#### 2.3.1. Determination of the Angle of Slide for Aerosil 200

The angle of slide for Aerosil 200 was measured by the following procedure.

Ten grams of Aerosil 200 were weighed accurately and placed at one end of a metal plate with a polished surface. This end was raised gradually until the plate made an angle with the horizontal surface at which the powder particles starts to slide. An angle of slide corresponds to 33° shown optimal flow properties [12].

#### 2.3.2. Determination of Flowable Liquid Retention Potential for Aerosil (*ϕ*-Value)

An increasing amount of liquid vehicle (PG, PEG400, and Tween 80) was added to the 10 g of Aerosil 200 powder and mixed well. The Aerosil adsorbed the liquid vehicle resulting into change in its flow properties. The angle of slide for aerosil was redetermined as stated earlier. The corresponding *ϕ*-value was calculated from the following equation:

(4) $$\begin{array}{c} \phi \text{-value}=\frac{\text{weight}\;\text{of}\;\text{liquid}}{\text{weight}\;\text{of}\;\text{solid}}. \end{array}$$

The *ϕ*-values were plotted graphically against the corresponding angle of slide which represented the flowable liquid retention potential of Aerosil.

According to Tayel et al. in propylene glycol, the *ϕ*-value is 0.16 for Avicel PH 102 and 1.5 for Aerosil 200. And for PEG 400, the *ϕ*-value is 0.005 for Avicel PH 102 and 3.26 for Aerosil 200, thus showing no need to determine it practically. The liquid load factor for PG and PEG 400 liquisolid system was calculated from flowable liquid retention potential using *R* value (excipient ratio) of 20, which is as follows [12]:

(5) $$\begin{array}{c} L_{f}=\phi +\phi \left(\frac{1}{R}\right),\\ L_{f}=0.16+1.5\left(\frac{1}{20}\right)=0.235,\\ L_{f}=0.005+1.5\left(\frac{1}{20}\right)=0.168. \end{array}$$

### 2.4. Precompression Studies of Liquisolid System

#### 2.4.1. Flow Properties of Liquisolid System

The flow properties of liquisolid system were estimated by determining the angle of repose, Carr's index, and Hausner's ratio. The angle of repose was measured by the fixed funnel method. The bulk density and tapped densities were determined for the calculation of Hausner's ratio and Carr's index [13, 14].

#### 2.4.2. Infrared Spectra Analysis

IR spectra of the liquisolid system were recorded by the KBr pellet method using the Fourier transform infrared spectrophotometer. A baseline correction was made using dried potassium bromide, and then the spectrum of pure RVT and liquisolid system was obtained.

#### 2.4.3. X-Ray Powder Diffraction (XRPD)

X-ray diffractograms of pure RVT and liquisolid formulation were obtained using Philips Analytical XRD instrument. The scanning range was from 5 to 80° at 2 theta scale.

#### 2.4.4. Differential Scanning Calorimetry (DSC)

Thermograms of the pure RVT and liquisolid system were recorded on a DSC. The thermal behaviour of the samples was investigated at a scanning rate of 10°C/min, covering a temperature range of 40–2600°C.

### 2.5. Post Compression Studies of Liquisolid Compacts

#### 2.5.1. Content Uniformity

In uniformity of drug content, 10 tablets from each batch were taken randomly to examine its content uniformity. Each tablet was weighed and crushed individually. The crushed tablet powders were dissolved in methanol. The solution was filtered using Whatman filter paper. The drug content was measured using UV spectrophotometer at 242.6 nm. According to the British Pharmacopoeia the percentages drug content of individual were calculated against the average drug content [15].

#### 2.5.2. Weight Variation

The weight variation test was performed on 20 tablets of liquisolid compacts as per British Pharmacopoeia [15].

#### 2.5.3. Hardness and Friability

The hardness of formulated liquisolid tablets was determined by using Pfizer hardness tester, and the mean hardness of the three liquisolid tablets was determined. The friability of the prepared liquisolid tablets was measured using Roche type apparatus, and the drum was rotated for 4 min at 25 rpm. The losses of the mass of 10 tablets were determined, and by using (6), the percentage of friability was calculated [16] as follow:

(6) $$\begin{array}{c} \%\;\text{Friability}=\left(\frac{\text{loss}\;\text{of}\;\text{mass}}{\text{Initial}\;\text{mass}}\right)\times 100. \end{array}$$

#### 2.5.4. Disintegration Test

 The disintegration test was performed at 37 ± 1°C in distilled water for six tablets from each formulation using the tablet disintegration unit. The tablets were considered completely disintegrated as no residue remains on the screen. Generally, ideal tablet hardness should be produced without applying excessive compression force where rapid tablet disintegration and drug dissolution are maintained at the same time [17]. 

#### 2.5.5. Wetting Time

Wetting is the important step for disintegration process to take place. A piece of tissue paper folded double was placed in a petri dish containing 6 mL of water. The tablet was placed on the paper, and the time for complete wetting of the tablet was measured in seconds. Wetting time corresponds to the time taken for the tablet to disintegrate when kept motionless on the tongue [18].

#### 2.5.6. *In Vitro* Dissolution Studies and Effect of Dissolution Volume on Drug Release Rate

*In vitro *dissolution profile from liquisolid compacts and directly compressed tablets was obtained using dissolution test apparatus USP-II. The dissolution studies were carried out in 900 mL, 450 mL, and 300 mL of 1.2 pH buffer and distilled water as the dissolution medium at 37°C ± 1°C and 50 rpm. Then, 5 mL samples were collected for up to 60 min at 5 min intervals up to 30 min and 15 min intervals from 30 to 60 min. The dissolution medium was replaced with 5 mL fresh dissolution fluid to maintain the sink condition. The withdrawn samples were filtered and analysed spectrophotometrically at 242.6 nm.

#### 2.5.7. Estimation of Fraction of Molecularly Dispersed Drug in Liquid Medication

 The fraction (*F*_*M*_) of the dissolved or molecularly dispersed drug in the liquid medication is the ratio of the drug's saturation solubility (*C*_*L*_) in the liquid vehicle (Table 2) over the drug concentration (*C*_*d*_) in the liquid medication:

(7) $$\begin{array}{c} F_{M}=\frac{C_{L}}{C_{d}}, \end{array}$$

where *F*_*M*_ = 1 when *C*_*L*_/*C*_*d*_ > 1.

 The value of fraction of the molecularly dispersed drug *F*_*M*_ (Table 1) in any system cannot exceed unity. 

## 3. Result and Discussion

### 3.1. Solubility Study

RVT was selected as the model drug for these studies, since it is a water-insoluble drug and thus an ideal candidate for testing the potential of rapid release liquisolid compact. In addition, it can be easily determined in solution using spectrophotometric principles and procedures. Beer's law was obeyed by all the standard curves of our RVT solution which were linear in the concentration range, that is, from 1 to 14 *μ*g/mL. The solubility of RVT in water, propylene glycol, polyethylene glycol 400, and polysorbate 80 (Tween 80) determined in these studies are shown in Table 2.

### 3.2. Precompression Studies of the Liquisolid System

#### 3.2.1. Flow Properties of the RVT Liquisolid System

The flow properties of the liquisolid powder system are influenced by physical, mechanical, and environmental factors. Therefore, different flow properties were employed. As the angle of repose (*θ*) is a characteristic of the internal friction or cohesion of the particles, the value of angle of repose will be high if the powder is cohesive and low if the powder is noncohesive. LS-1 shows a good flow property with *θ* value of 29.59 and is considered as a liquisolid system with acceptable flowability. Carr's index up to 16 was considered acceptable as a flow property. Hausner's ratio was related to the interparticle friction; powders with a low interparticle friction had a ratio of approximately 1.25 indicating a good flow. The LS-1 system with Carr's index of 14.63 and Hausner's ratio of 1.17 was considered for further study, and results of all batches of RVT liquisolid compacts are shown in Table 3.

#### 3.2.2. IR Spectra Analysis

**Figure 1:**
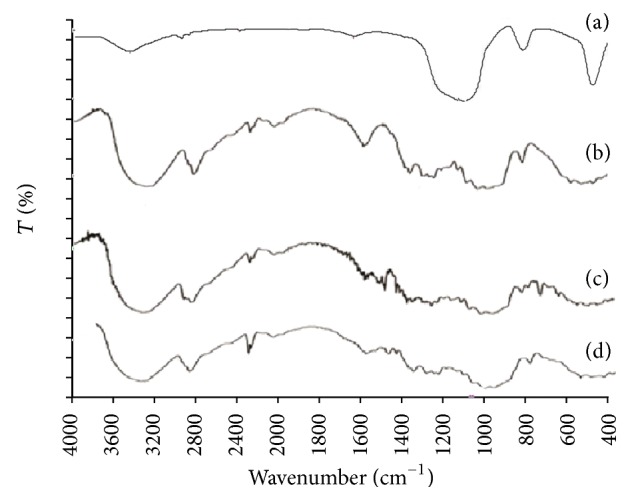
FT-IR spectra of (a) Aerosil 200, (b) Avicel PH 102, (c) pure RVT, and (d) liquisolid system (LS-1).

Samples of Aerosil 200, Avicel PH-102, pure RVT, and liquisolid system (LS-1) were subjected to FT-IR spectroscopic analysis, and their spectra at 500–4000 cm^−1^ are shown in Figure 1. Characteristics peaks of aromatic N-H stretching and C=O stretching at 3364.57 cm^−1^ and 1556.55 cm^−1^ appeared, respectively. A reduction in the intensity of the characteristics absorption bands of RVT was observed in the liquisolid formulation, which might be attributed to the hydrogen bonding interaction between the carboxylic group of RVT and the hydroxyl group of the liquid vehicles; this resulted in drug dissolution enhancement. 

#### 3.2.3. X-Ray Powder Diffraction (XRPD)

**Figure 2:**
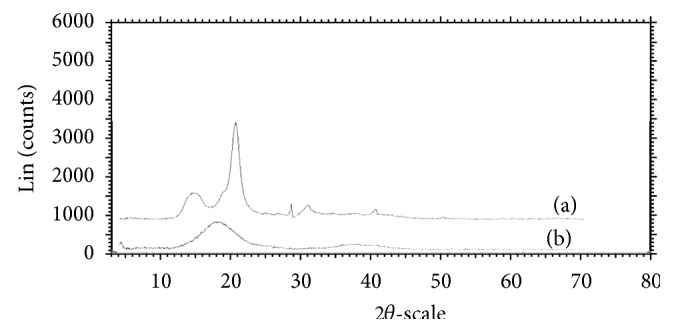
X-ray diffractogram of (a) pure drug RVT and (b) liquisolid system LS-1.

Polymorphic changes in the drug are important since they might affect the dissolution rate and in-line bioavailability. Hence, it was necessary to study the polymorphic changes of RVT in liquisolid compacts.  Figure 2 shows XRPD of pure RVT (a) and liquisolid system (b). X-ray diffraction pattern in Figure 2 demonstrated that pure RVT was clearly in crystalline state as it showed sharp distinct peaks at 2*θ* diffraction angles of 15.5°, 22.6°, 31.7°, 34.5°, and 45.5°. XRPD pattern of formed liquisolid showed absence of sharp peaks. Such an absence of RVT constructive reflection (specific sharp peak) in the liquisolid X-ray diffractogram indicates that RVT has almost entirely converted from crystalline to amorphous or solubilised form; such lack of crystallinity in the liquisolid system was understood to be a result of RVT solubilisation in the liquid vehicle that was absorbed into and adsorbed onto the carrier and coating material.

#### 3.2.4. Differential Scanning Calorimetry (DSC)

**Figure 3:**
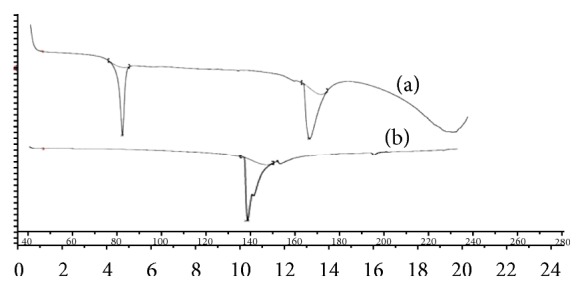
DSC thermograms of (a) pure RVT and (b) liquisolid system LS-1.

The possible interaction between a drug entity and excipient in liquisolid compact was determined by DSC.  Figure 3 shows thermal behaviour of the pure component (a) together with the thermal behaviour of the formulated liquisolid system (b). Pure RVT shows two characteristic peaks at 80°C and 164°C this is because of the polymorphic form of RVT, that is, form “S”, and it is a primary indication for crystalline nature of pure drug. Furthermore, thermal behaviour of liquisolid system (LS-1) shows the shifting of peak at 143°C. It indicates that crystalline nature of drug gets completely converted into amorphous form due to which there is a significant change in endothermic peak of formed liquisolid system. 

### 3.3. Post Compression Studies of Liquisolid Compacts

#### 3.3.1. Content Uniformity

All the liquisolid and conventional tablets complied with the British Pharmacopoeia (BP) weight uniformity test; also, all the tablets had met content uniformity criteria, as per BP, in which each individual content was between 85% and 115% of the average content.

#### 3.3.2. Friability, Hardness, Weight Variation, Disintegration Test, and Fraction of Molecularly Dispersed Drug (*F*_*M*_)

All the RVT liquisolid tablets exhibit the acceptable friability. The percentage did not exceed 1% of the tablet weight, and no tablet was broken or deformed. Since all the prepared formulae met the standard friability criteria, they are expected to show acceptable toughness and withstand abrasion during handling. All the prepared batches had hardness in range 3–5 kg/cm^2^. Generally, the ideal tablet hardness should be produced without applying excessive compression force where rapid tablet disintegration and drug dissolution are maintained at the same time [16]. Also, the batches passed the USP weight variation test. All the prepared batches had a disintegration time less than 1 min. The batches prepared with increasing drug concentration exhibited an increasing disintegration time. Based on (6), *F*_*M*_ value of each liquisolid preparation was calculated. No liquid vehicle is involved in the case of directly compressed tablets which contain plain micronized RVT powder, and their *F*_*M*_ value was taken as 0. The result of friability, hardness, weight variation, disintegration time, and fraction of molecularly dispersed drug (*F*_*M*_) for all the batches of RVT liquisolid compacts are shown in Table 4.

#### 3.3.3. Wetting Time

**Figure 4:**
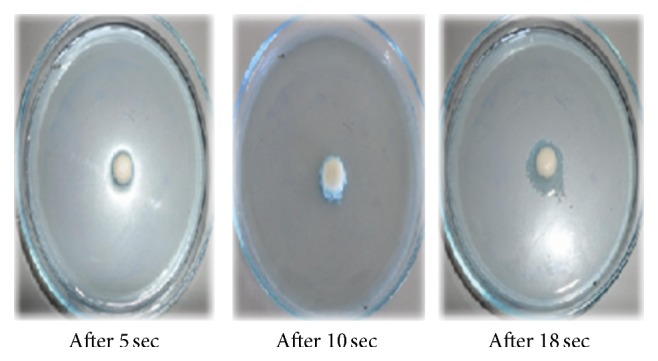
The wetting time of liquisolid system (LS-1) in seconds.

Wetting time is closely related to the inner structure of the tablets and to the hydrophilicity of the excipient. A linear relationship exists between wetting time and disintegration time. The wetting time of liquisolid system (LS-1) was found to be 18–20 sec as compared to the conventional tablet which showed wetting time as 35–38 sec. The wetting time for liquisolid system (LS-1) was shown in Figure 4.

#### 3.3.4. *In Vitro* Dissolution Studies and Effect of Dissolution Volume on Drug Release Rate

**Figure 5:**
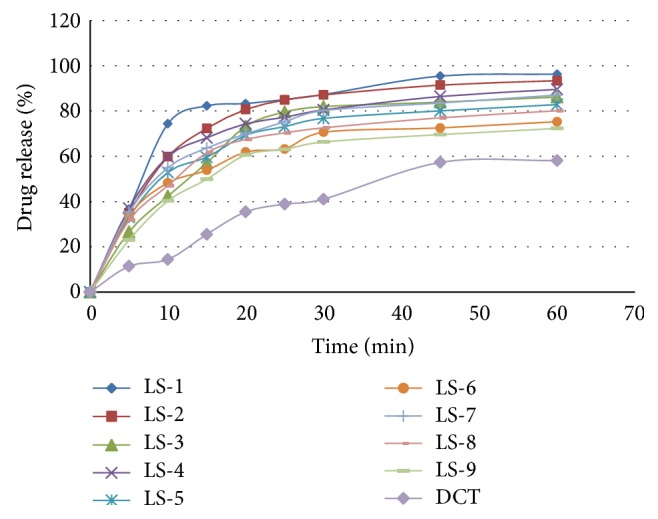
Dissolution profile of RVT liquisolid compacts and direct compressed tablet in 1.2 pH.

**Figure 6:**
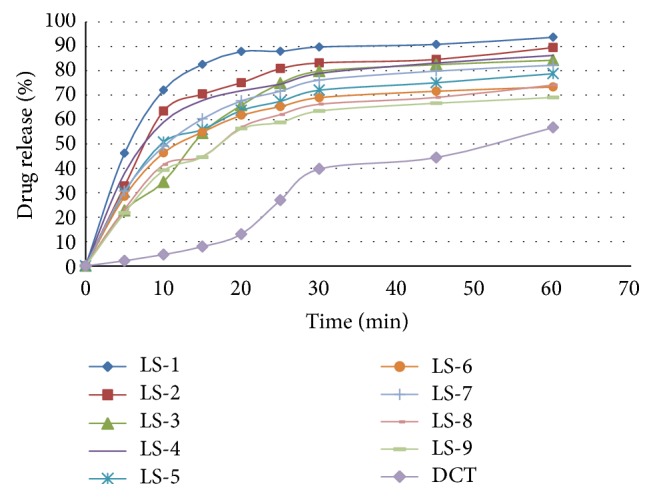
Dissolution profile of RVT liquisolid compacts and directly compressed tablet in distilled water.

Figures 5 and 6 showed the dissolution profile of liquisolid compact of LS-1 to LS-9 with direct compressible tablet in 1.2 pH and distilled water. Liquisolid compact LS-1 produced a higher dissolution in 1.2 pH and distilled water in comparison with conventional tablet. 

**Figure 7:**
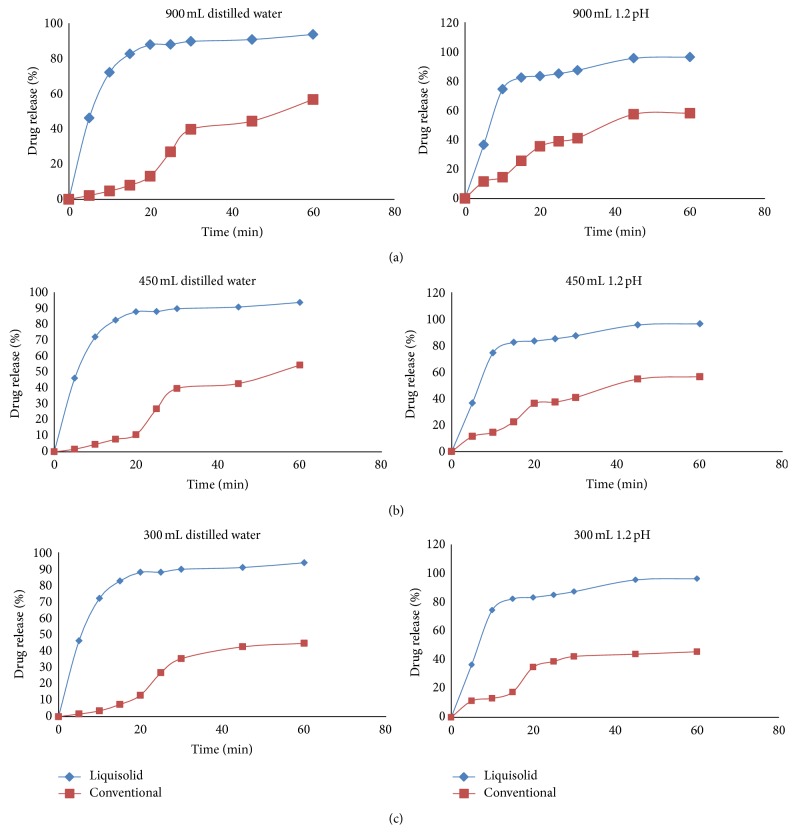
Comparison of dissolution profile displayed by the liquisolid compact (LS-1) and conventional tablet in 900 mL, 450 mL and 300 mL volume of distilled water and 1.2 pH.

Figure 7 shows the drug dissolution profiles from the liquisolid compacts (LS-1) and the directly compressed tablets (DCT-1) of micronized RVT calcium. When 900 mL (per vessel) of distilled water or 1.2 pH solution was used as the dissolving medium, liquisolid tablets displayed slightly better *in vitro* release characteristics than those of their directly compressed counterparts.

However, when smaller volumes, such as 450 and 300 mL, of dissolution media's were used, the liquisolid tablets demonstrated significantly improved drug dissolution properties.

**Figure 8:**
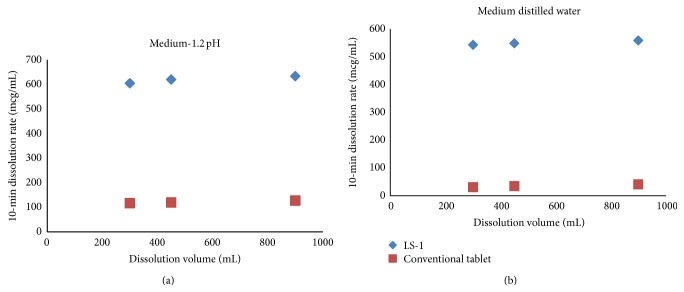
Effect of the dissolution volume on the initial dissolution rate of liquisolid compact (LS-1) exhibited by the liquisolid compacts and directly compressed tablets using different dissolution media.

It seems that the drug dissolution rate of liquisolid compacts is significantly faster than that of the plain tablets, and it is independent of the volume of the dissolving liquid used. Furthermore, it is apparent that decreasing dissolution volumes result in a proportional decrease of the *in vitro* drug release rates displayed by the directly compressed tablets. According to the “diffusion layer model” dissolution theories, the dissolution rate of a drug is directly proportional to its concentration gradient (Δ*C* = *C*_*s*_ − *C*) in the stagnant diffusion layer formed by the dissolving liquid around the drug particles. *C*_*s*_ is the saturation solubility of the drug in the dissolution medium, and, thus, it is a constant characteristic property related to the drug and dissolving liquid involved. On the other hand, *C*, the drug concentration in the bulk of the dissolving medium, increases with decreasing volumes of dissolution fluid used. Therefore, the Δ*C* values existing in the three different dissolution volumes of our tests decrease with decreasing volumes of dissolution medium. Consequently such Δ*C* reduction is directly related to the decreased drug dissolution rates of the conventional tablets with the decreasing volumes of both the dissolution media used (Figure 8).

## 4. Conclusion

The overall objective of present study was to enhance dissolution of poorly water-soluble RVT by liquisolid compact technique. The liquisolid tablets formulated with the propylene glycol at drug concentration of 10% w/w is the best formulation among all the batches of liquisolid tablets prepared, in terms of faster disintegration time, superior dissolution profile, and acceptable tablet properties. Propylene glycol was found to be a promising liquid vehicle in formulating liquisolid formulations of RVT calcium. The liquid vehicle plays a contributing role in improving the dissolution profiles of a poor water-soluble drug in the liquisolid formulations, besides choosing a suitable liquid vehicle according to its viscosity and HLB value. The key step in formulating a successful liquisolid tablet is the determination of the optimal flowable liquid retention potential (*ϕ*-value). 

## Figures and Tables

**Table 1:** Key formulation characteristics of prepared RVT liquisolid tablets.

Liquisolid system	Liquid vehicle	Drug concentration (% w/w) in liquid medication (*C*_*d*_)∗	Excipient ratio (*R*)^†^	Avicel PH-102 (*Q*)^‡^ mg	Aerosil 200 (*q*)^*∧*^ mg	Liquid load factor (*L*_*f*_)^#^	Molecular fraction (*F*_*M*_)^+^
LST-1	PG	10	20	166.6	8.33	0.235	1.074
LST-2	PG	15	20	249.9	12.49	0.235	0.716
LST-3	PG	20	20	333.2	16.66	0.235	0.537
LST-4	PEG-400	10	20	227.1	11.35	0.168	0.452
LST-5	PEG-400	15	20	343.0	17.15	0.168	0.301
LST-6	PEG-400	20	20	454.3	22.71	0.168	0.226
LST-7	Tween-80	10	20	287.7	14.38	0.195	0.348
LST-8	Tween-80	15	20	431.5	21.57	0.195	0.232
LST-9	Tween-80	20	20	575.3	28.76	0.195	0.174

^*^An appropriate amount of liquid medication containing 10 mg of drug was incorporated in each tablet.

^†^Excipient ration (*R*) equal to 20 in each formulation.

^‡^Amount of carrier (*Q*) material.

^*∧*^Amount of coating (*q*) material.

^#^Liquid load factor is defined as *L*_*f*_ = *W*/*Q*.

^+^The fraction (*F*_*M*_) of molecularly dispersed drug was calculated on the basis of (7).

**Table 2:** Solubility of RVT in different solvents.

Solvent	Solubility (% w/w)
	(*C*_*L*_)
Distilled water	0.0125
Propylene glycol	10.7481
Polyethylene glycol 400	4.5286
Polysorbate 80 (Tween 80)	3.4814

*C*_*L*_: the saturation solubility of drug in non-volatile liquid vehicle.

**Table 3:** Flow properties of RVT liquisolid systems.

Liquisolid system	Angle of repose	Bulk density (gm/cm^3^)	Tapped density (gm/cm^3^)	Carr's index	Hausner's ratio
LS-1	29.59 ± 0.51	0.24 ± 0.01	0.28 ± 0.02	14.63 ± 0.21	1.17 ± 0.01
LS-2	30.28 ± 0.24	0.23 ± 0.02	0.28 ± 0.02	16.69 ± 0.32	1.20 ± 0.01
LS-3	31.50 ± 0.32	0.23 ± 0.02	0.27 ± 0.01	15.30 ± 0.15	1.18 ± 0.04
LS-4	30.45 ± 0.45	0.28 ± 0.04	0.33 ± 0.04	15.69 ± 0.53	1.18 ± 0.01
LS-5	30.83 ± 0.22	0.23 ± 0.01	0.28 ± 0.08	17.87 ± 0.12	1.21 ± 0.02
LS-6	33.86 ± 0.43	0.25 ± 0.03	0.31 ± 0.01	19.00 ± 0.14	1.23 ± 0.01
LS-7	32.82 ± 0.55	0.27 ± 0.02	0.32 ± 0.03	16.25 ± 0.42	1.19 ± 0.05
LS-8	33.86 ± 0.24	0.27 ± 0.02	0.33 ± 0.15	18.05 ± 0.53	1.22 ± 0.04
LS-9	36.72 ± 0.25	0.25 ± 0.04	0.31 ± 0.05	20.25 ± 0.12	1.25 ± 0.01
DCT	37.77 ± 0.43	0.28 ± 0.01	0.34 ± 0.02	18.32 ± 0.24	1.22 ± 0.01

**Table 4:** Physical parameter of liquisolid tablet.

Liquisolid system	Hardness (kg/cm^2^)	Thickness (mm)	Disintegration time (sec)	Friability (%)	Weight variation (mg)	Content uniformity (%)
LS-1	3.82 ± 0.28	3.4 ± 0.02	14 ± 0.28	0.21	241.8 ± 1.1	99.25 ± 0.24
LS-2	4.20 ± 0.42	3.0 ± 0.01	16 ± 0.51	0.30	253.4 ± 0.8	98.25 ± 0.20
LS-3	4.62 ± 0.31	2.9 ± 0.01	18 ± 0.50	0.45	341.5 ± 1.8	98.00 ± 0.51
LS-4	3.91 ± 0.14	3.4 ± 0.02	17 ± 0.32	0.33	272.1 ± 1.2	96.51 ± 0.44
LS-5	4.42 ± 0.31	3.2 ± 0.04	22 ± 0.40	0.45	368.4 ± 1.6	97.26 ± 0.23
LS-6	4.71 ± 0.15	3.0 ± 0.02	26 ± 0.14	0.53	483.1 ± 0.4	98.60 ± 0.45
LS-7	4.15 ± 0.18	3.7 ± 0.01	19 ± 0.21	0.29	332.5 ± 1.0	98.21 ± 0.50
LS-8	4.54 ± 0.35	3.4 ± 0.03	24 ± 0.53	0.37	492.2 ± 0.7	95.27 ± 0.15
LS-9	4.62 ± 0.23	2.9 ± 0.01	28 ± 0.42	0.34	638.1 ± 1.5	97.51 ± 0.25
DCT	4.80 ± 0.22	3.5 ± 0.01	44 ± 0.15	0.48	248.4 ± 0.5	99.00 ± 0.42
